# A Case of Complete Renal Duplex with H-Shaped Ureter

**DOI:** 10.1155/2012/643207

**Published:** 2012-06-07

**Authors:** A. Ghobashy, M. El-Shazly, A. Lari, O. Al-Hunaidy, A. Allam, N. Alenezy, E. Yordanov, B. Hathout

**Affiliations:** Urology Department, Farwaniya Hospital, P.O. Box 482, Ardiya 92400, Kuwait

## Abstract

We present a case of complete renal duplex with H-shaped double ureter opening into the bladder with 2 separate orifices. It is an extremely rare variety of renal duplex which was reported only once in the literature. Fifty-four-year-old male presented to our department with right renal pain. Noncontrast CT revealed stone midthird right ureter with duplex right kidney. Retrograde ureteropyelography and ureteroscopy revealed this rare anomaly.

## 1. Introduction

The prevalence of double ureter ranges from 0.1% to 3%, as reported by various author [1, 2]. The detailed anatomy of renal duplex and detection of site of ureteric insertion are often difficult to be determined accurately preoperatively [2, 3].

## 2. Case Report

**Figure 1:**
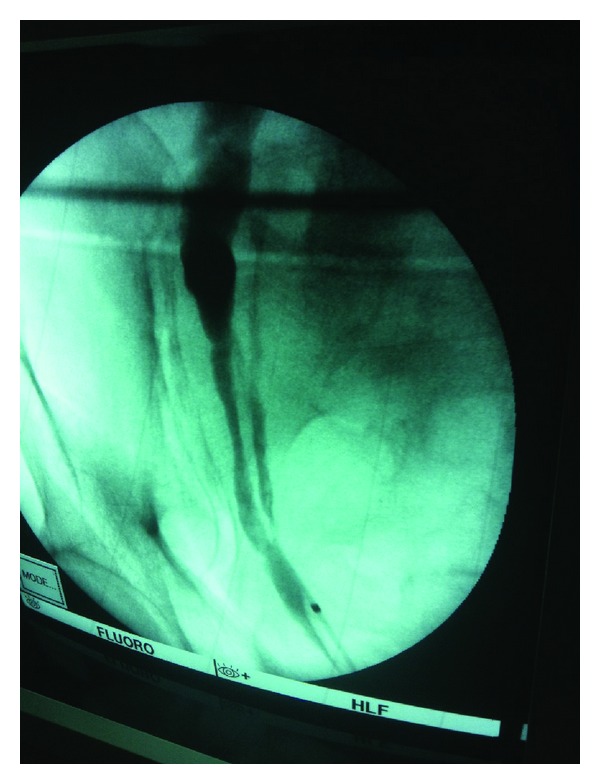
Retrograde pyelography showing H-shaped ureter.

Fifty-four-year-old patient presented to our department with recurrent right loin pain. KUB was nonremarkable. Non-contrast CT revealed 1 cm stone in the middle third of right ureter with hydronephrosis of upper moiety of right renal duplex. Diagnostic cystoscopy revealed 2 separate ureteric orifices on the right side of the trigone suggesting double ureter (Figure 1). Retrograde uretero-pyelography revealed H-shaped ureter. Retrograde uretero-pyelography was performed through injecting contrast through ureteric balloon dilator inserted in one orifice. Another balloon dilator was inflated in the other orifice. This was technically very helpful as the balloon prevented slippage of contrast down to the bladder due to proximity of the junction between the two ureters from the urinary bladder. Ureteroscopy revealed union of the 2 ureters about 2 cm from the bladder for a short distance about 1 cm then the two ureters separate again up to the duplex kidney. Ureteroscope was advanced easily to the site of the stone as the junction of the 2 ureters was wide enough for passage of the ureteroscope. Stone disintegration was performed using pneumatic lithoclast. Retrieval of the fragments was carried out using forceps.

## 3. Discussion

It is well reported in the literature that double ureter and duplex system have potential for future complications, such as obstructive uropathy, stone formation, ureterocele, and vesicoureteral reflux. This makes early detection of this anomaly helpful to prevent comorbidities and complications [4].

Reviewing the literature, H-shaped ureter was reported only once [5].

Complete renal duplex with double ureters opening separately into the urinary bladder can be embryologically explained as a development of two ureteral buds separately from a single mesonephric duct [6].

The 2 separate ureteric buds can fuse for a short distance near their exit from the urinary bladder then separate again explaining embryologically this rare anomaly.

## 4. Conclusion

Congenital anomalies of the urinary tract as duplex kidney and double ureter should be suspected and promptly detected to avoid the possible complications. Although it is rare, H-shaped ureter should be in mind.
